# Cardiac spheroids as promising *in vitro* models to study the human heart microenvironment

**DOI:** 10.1038/s41598-017-06385-8

**Published:** 2017-08-01

**Authors:** Liudmila Polonchuk, Mamta Chabria, Laura Badi, Jean-Christophe Hoflack, Gemma Figtree, Michael J. Davies, Carmine Gentile

**Affiliations:** 1 Roche Pharma Research and Early Development, Roche Innovation Center Basel, F. Hoffmann-La Roche Ltd., Basel, 4070 Switzerland; 2 Sydney Medical School, University of Sydney, Sydney, 2000 Australia; 3 Department of Biomedical Sciences, Panum Institute, University of Copenhagen, Copenhagen, 2200 Denmark; 4 Heart Research Institute, Newtown, 2041 Australia; 5 Beth Israel Deaconess Medical Center, Harvard Medical School, Boston, USA

## Abstract

Three-dimensional *in vitro* cell systems are a promising alternative to animals to study cardiac biology and disease. We have generated three-dimensional *in vitro* models of the human heart (“cardiac spheroids”, CSs) by co-culturing human primary or iPSC-derived cardiomyocytes, endothelial cells and fibroblasts at ratios approximating those present *in vivo*. The cellular organisation, extracellular matrix and microvascular network mimic human heart tissue. These spheroids have been employed to investigate the dose-limiting cardiotoxicity of the common anti-cancer drug doxorubicin. Viability/cytotoxicity assays indicate dose-dependent cytotoxic effects, which are inhibited by the nitric oxide synthase (NOS) inhibitor L-NIO, and genetic inhibition of endothelial NOS, implicating peroxynitrous acid as a key damaging agent. These data indicate that CSs mimic important features of human heart morphology, biochemistry and pharmacology *in vitro*, offering a promising alternative to animals and standard cell cultures with regard to mechanistic insights and prediction of toxic effects in human heart tissue.

## Introduction

Engineering of three-dimensional myocardial tissues presents several advantages for cultures of primary cardiomyocytes (CMs) to study human heart biology, physiology and pharmacology^1–3^. In contrast to monolayer cultures, CMs cultured in a three-dimensional environment have prolonged viability and can retain their contractile properties. For instance, CMs cultured in a three-dimensional alginate matrix retain their sarcomeric structure for up to 14 days in contrast to a few days in a monolayer culture^4^. Similarly, Chan *et al*.^5^ showed that neonatal rat CMs retain their contractile properties when cultured in a three-dimensional multi-strip cardiac muscle containing fibrinogen and Matrigel. Co-cultures of CMs with other cell types, such as endothelial cells (ECs) and cardiac fibroblasts (CFs), also improve engraftment and functional features in an *in vivo* myocardial infarction (MI) model compared to sheets of CMs alone^6^.

As primary cardiomyocyte sources are limited, stem cell-derived CMs have emerged as a promising cell source to model the human heart, with obvious advantages for toxicity and drug discovery studies, and regenerative therapy^7, 8^. One of the main limitations of human pluripotent stem cell-derived CMs or cardiac progenitor cells is their immature phenotype, with these having contractile and other biological and physiological properties that differ from those of human adult CMs^9–12^. Although Fernandes *et al*.^13^ have shown that stem cell-derived CMs and cardiac progenitor cells of different sources have similar regenerative properties in an *in vivo* myocardial infarction model in mice, a deeper understanding of the requirements for optimal engineering of a human heart model *in vitro* is important^14^. Approaches include use of different cell sources and methods that alter the phenotype from immature to adult CMs. Scaffold-based and scaffold-free approaches can be applied to engineer the heart^12, 15^. However, most of the cardiac tissue models contain only CMs and lack the other cell types present in the human heart, such as ECs and CFs. Caspi *et al*.^16^ have generated vascularized cardiac tissue by seeding ESC-derived CMs, ECs and CFs into a scaffold. This approach has considerable advantages as pre-vascularization of cardiac tissues has been shown to be crucial in determining therapeutic outcomes^17^.

In this study we developed a 3D *in vitro* model of the human heart microenvironment using ECs, CFs and adult CMs (the *human cardiac spheroid*, *hCS*) in proportions similar to those found in the human heart. A similar approach has been used to develop a 3D co-culture from commercially available iPSC-derived cells (*iPSC-derived cardiac spheroid*, *iCS*). These models have been used to elucidate the mechanism of cardiotoxicity of the widely-used anti-cancer drug, doxorubicin (DOX; also named adriamycin) which can modulate nitric oxide (NO) levels.

Constitutive synthesis of NO in the heart has been associated with a cardioprotective role played by this molecule in both an autocrine^18, 19^ and paracrine^20^ manner (*via* ECs). While low concentrations of NO have cardioprotective effects^21^, increased levels of NO associated with oxidative stress and peroxynitrous acid generation, have been suggested to be responsible for the undesired cardiotoxic effects of anthracyclines, such as DOX^22–25^ dramatically limiting their use as chemotherapeutic agents^26–29^. Among the different proposed mechanisms, reactive oxygen species (ROS) seem essential for mediating anthracycline-induced cardiotoxicity and DNA damage following its subcellular accumulation^30–35^. Recent *in vitro* and *in vivo* data suggest an alternative mechanism to target DOX/ROS-mediated cardiotoxic effects, which may involve NO synthases (NOS)^36–41^. This led us to investigate if, and how, NO mediates DOX-induced cardiotoxic effects in human heart cells, and the dual cardioprotective and cardiotoxic role of this species, which highlights the importance of cellular crosstalk.

## Results

### Generation of cardiac spheroids from primary and iPSC-derived cardiac cells

**Figure 1:**
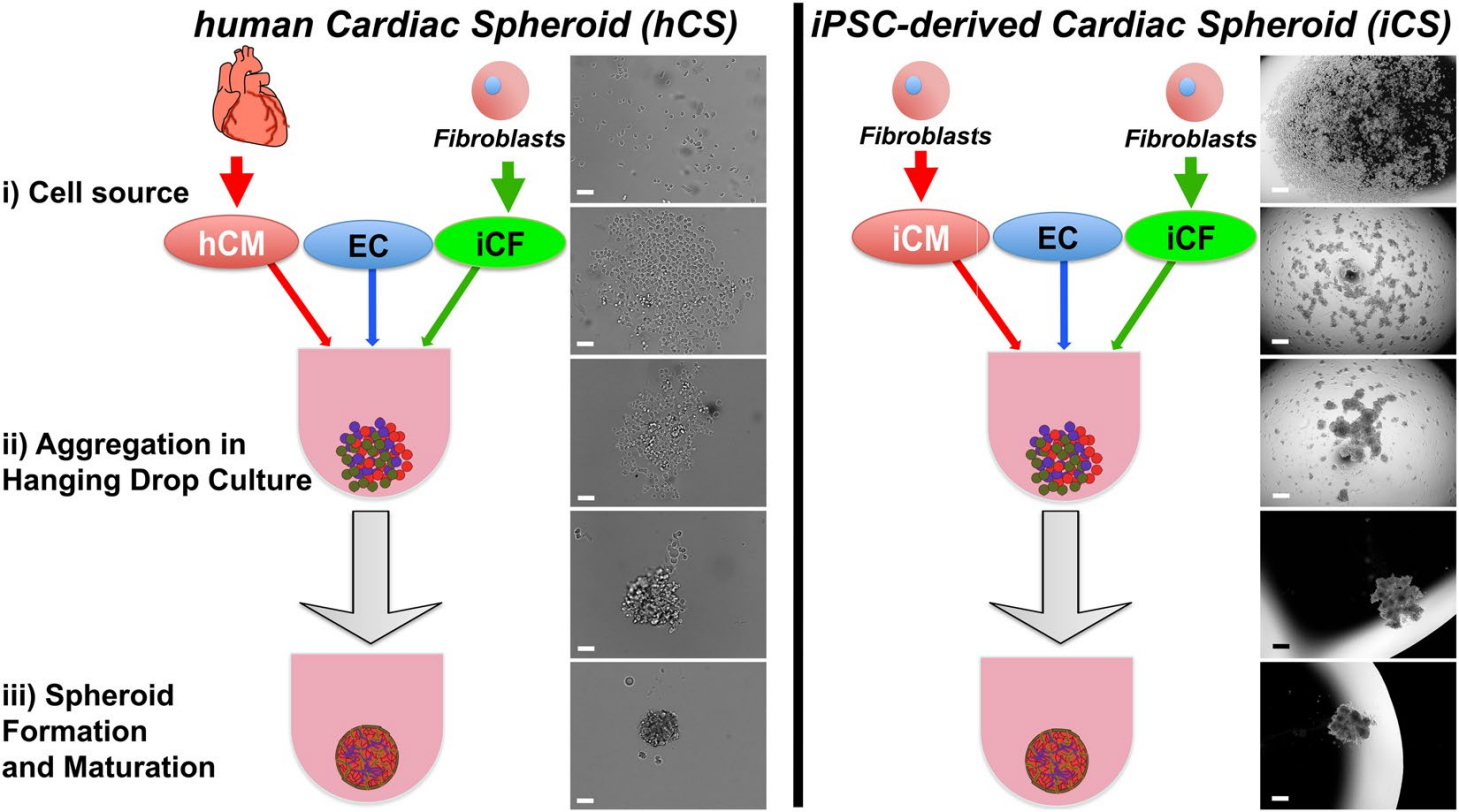
Generation of cardiac spheroids in hanging drop culture. (**a**) Representative bright-field images of human primary cardiomyocytes (hCM), iPS-derived cardiac fibroblasts (iCF) and human coronary artery endothelial cells (ECs) co-cultured in hanging drops for 0, 2, 6, 12 and 24 h. (**b**) Representative bright-field images of hiPS-derived cardiomyocytes (iCM), iCF and ECs in hanging drop co-cultures after 0, 12, 24, 48 and 96 h. Scale bars: 100 μm. See also Figs S1, S2 and Videos S1 and S2.

To investigate the roles played by the microenvironment in the human heart we have developed a 3D *in vitro* “*cardiac spheroid*” model of the human heart. Hanging drop cultures of cells normally found within the human heart were prepared using human coronary artery endothelial cells (ECs), cardiac fibroblasts derived from iPSCs (iCFs) and either human primary adult cardiomyocytes (hCMs) or iPSC-derived cardiomyocytes (iCMs, Fig. 1). hCMs were isolated from frozen heart sections, retaining the biochemical, morphological and physiological features of fresh human heart tissue (Fig. S1a–c). Similar human heart sections were used to compare the expression levels of markers in spheroids and *ex vivo*.

These spheroids have been named according to their cell population: human (*hCSs*) and iPSC-derived cardiac spheroids (*iCSs*). Due to differences in cell size between hCMs and iCMs (approximately 150 μm and 50 μm in length, respectively) and the limited viability of hCMs in culture, we employed different cell ratios to mimic the *in vivo* cardiac microenvironment and to support spheroid formation. We increased the number of iCFs and ECs to maintain hCMs in culture, with a ratio of 1:3:6 for hCM:EC:iCF, respectively. iCMs were co-cultured in a ratio of 2:1:1 for iCM:EC:iCF, respectively. The different cell types and ratios affected the kinetics of CS formation, with hCSs forming aggregates within 24 h due to the higher numbers of iCFs, whereas iCSs with less iCFs required four days to completely aggregate in hanging drop cultures (Fig. 1). TEM analysis showed that hCSs retain their sarcomeric structure in these 3D *in vitro* cultures (Fig. S2). To elucidate the role of individual cell types, spheroids were also generated containing one or two of the cell types. Hanging drop cultures of hCMs alone did not aggregate and eventually died as indicated by the presence of cell debris after 2 weeks in culture, whereas iCMs aggregated, but formed loose non-spherical clusters (data not shown). Co-cultured hCMs and ECs formed small aggregates but these did not coalesce into a single large spheroid (data not shown). Following spheroid formation, iCSs exhibited synchronous contraction in hanging drop cultures (see Videos S1 and S2 “mini beating heart”) demonstrating that these multiple-cell type CSs have structural and functional characteristics representative of human heart tissue.

### Cardiac fibroblasts and endothelial cells control the three-dimensional behavior of cardiac myocytes

**Figure 2:**
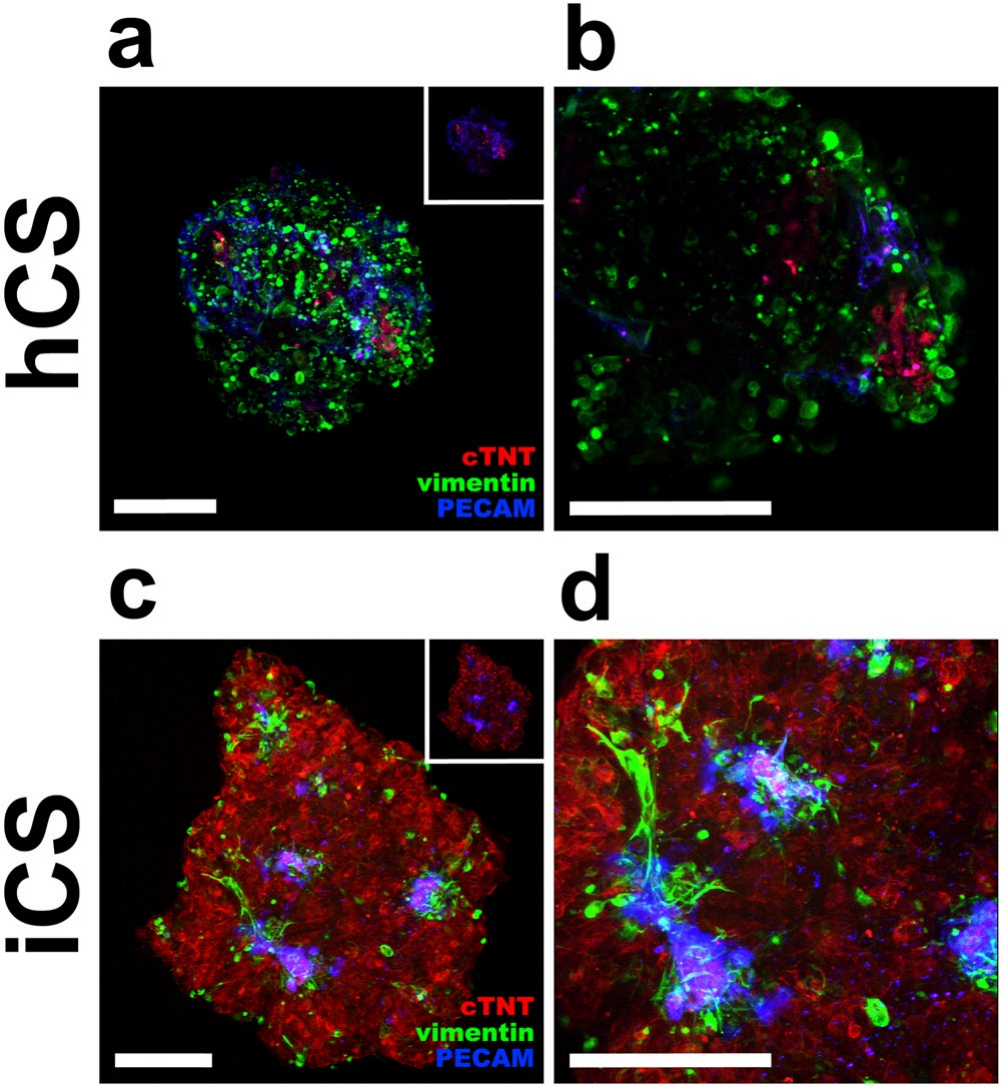
Sorting of cardiomyocytes, endothelial cells and fibroblasts within cardiac spheroids. (**a**–**d**) PECAM-positive ECs (blue) form a microvascular network within hCSs (**a**,**b**) and iCSs (**c,d**) in hanging drop cultures. Vimentin-positive iCFs (green) sort out both on the surface and within hCSs (**a**,**b**) and iCSs (**c,d**). Cardiac Troponin T-positive iCMs (red) are surrounded by both ECs and iCFs in hCSs (**a,b**) and iCSs (**c,d**). Scale bars: 100 μm in (**a,b**), 200 μm in (**c,d**). See also Fig. S3.

The biochemical properties of these cardiac spheroids were investigated by immuno-staining using antibodies against markers for CMs (cardiac Troponin T), CFs (vimentin) and ECs (PECAM). Confocal analysis showed that both hCSs and iCSs have a significant vascular network (Fig. 2a and c). The inserts in Fig. 2a and c show that ECs line the vascular network, with CMs in closely proximity, as observed in human tissue sections. iCFs are present both on the surface and throughout the spheroid, in support of the developing vascular network of both hCSs and iCS. Analysis of iCSs showed that vimentin-positive iCFs are adjacent to developing PECAM-positive microvascular network (Fig. S3a–c). Imaris 3D rendering analysis of iCSs showed that iCFs alone define a supporting structure for this network that allows vascularization throughout the spheroid (Fig. S3d–f).

**Figure 3:**
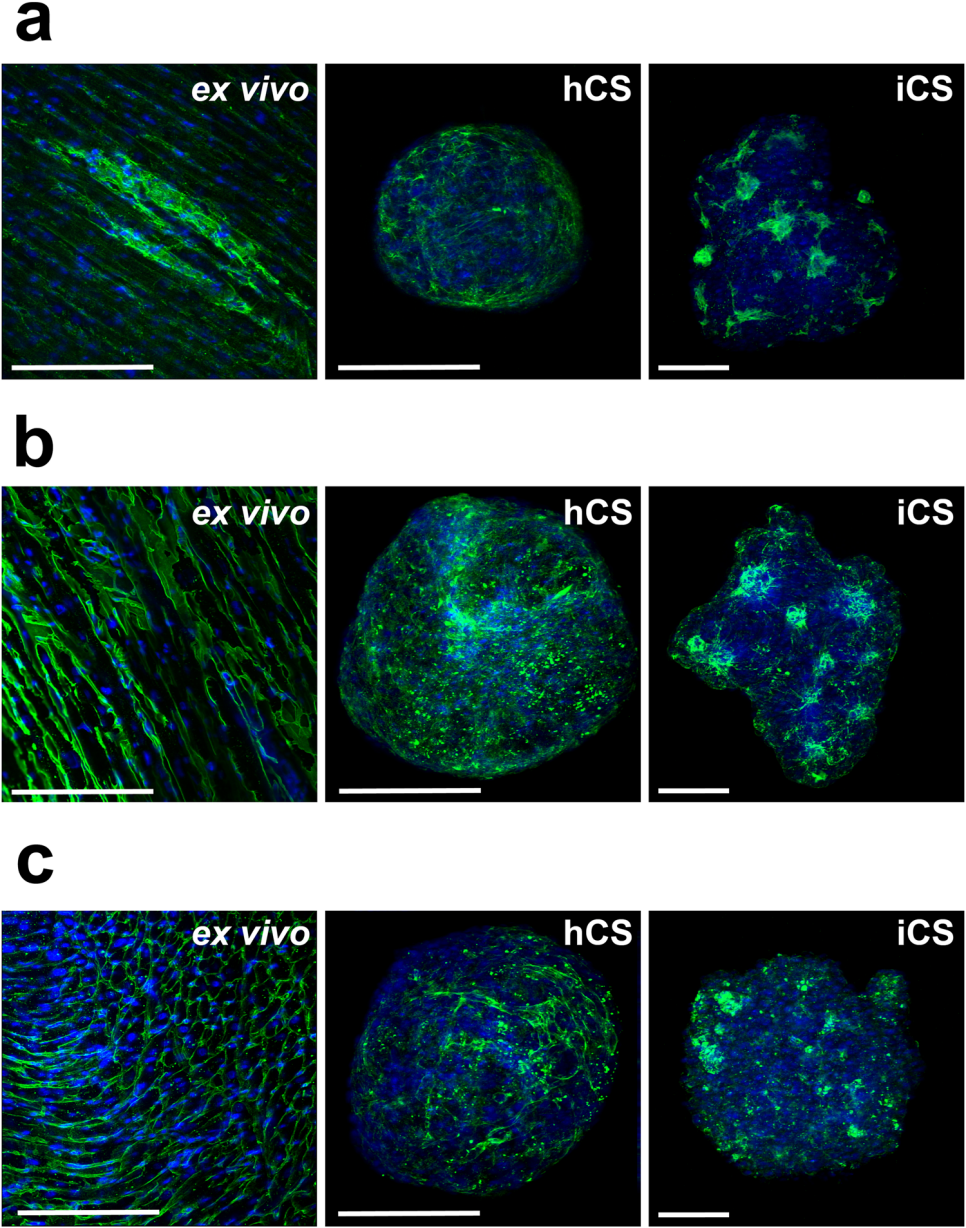
Extracellular matrix (ECM) deposition *ex vivo* and *in vitro*. Fibronectin (**a**), collagen type IV (**b**) and laminin (**c**) deposition within human heart sections, hCSs and iCSs (green). Nuclei are stained with Hoechst stain (blue). Scale bars: 200 μm.

The deposition of ECM molecules in both hCSs and iCSs was also examined. Analysis of confocal images of *ex vivo* heart sections, hCSs and iCSs showed similarities to both *in vitro* models and human heart tissue (Fig. 3), with a network of fibronectin, collagen type IV and laminin present in each case. The more regular organisation in hCS may be due to the higher numbers of CMs in the iCSs. ECM deposition was distributed within tissues according to the sorting of ECs and iCFs (Fig. 2).

Overall, these data demonstrate that both hCSs and iCSs mimic the cellular and molecular composition of human heart tissue.

### Doxorubicin-mediated toxic effects on hCSs and iCSs

**Figure 4:**
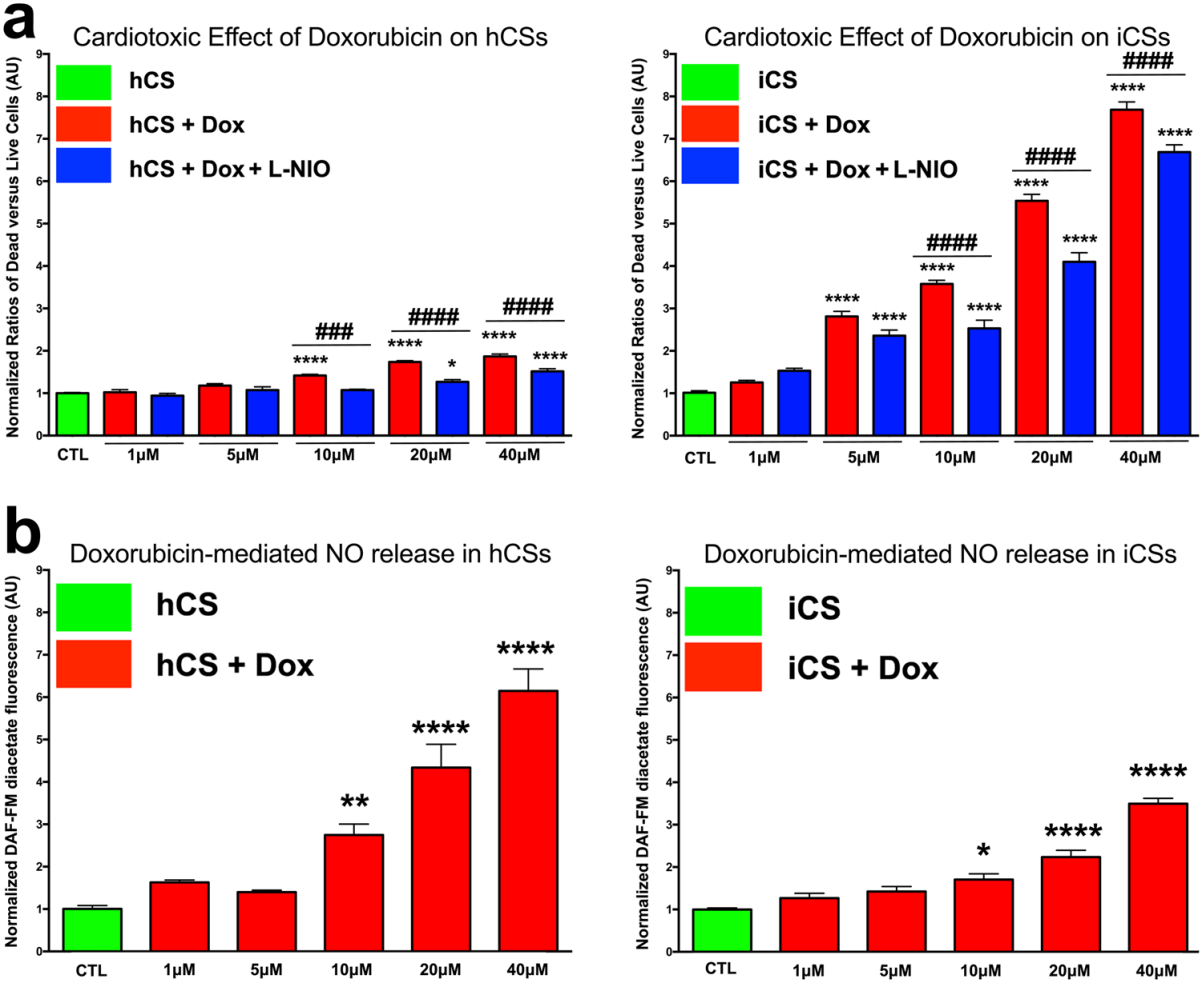
Inhibition of nitric oxide synthesis prevents DOX-mediated cardiotoxic effects in hCSs and iCSs. (**a**) Statistical analysis (*n* = 6) of DOX-mediated cardiotoxic effects in hCSs and iCSs evaluated as a ratio between dead and live cells after 24 h treatment. 100 μM L-NIO prevents the doxorubicin-mediated effects in both hCSs and iCSs treated with 10, 20 and 40 μM doxorubicin. (**b**) Statistical analysis (*n* = 6) of DAF-FM-positive cells in hCSs and iCSs following 24 h doxorubicin treatment. *Relative to control for all groups; ^♯^Within the same concentration. Data are presented as mean ± SEM. One-way ANOVA followed by Bonferroni’s multiple comparisons test. F (df 10, 55)=40.53 for (**a**) hCS, F (df 10, 143) = 253 for (**a**) iCS, F (5, 30) = 37.33 (**b**) hCS and F (5, 30)=56.09 for (**b**) iCS. See also Fig. S4.

To evaluate if hCSs and iCSs could be used as *in vitro* models to study human heart toxicology, they have been treated with increasing concentrations of a known cardiotoxic agent, doxorubicin (DOX). To evaluate its toxic effects on hCSs and iCSs, we have measured dead versus live ratios in controls, and hCSs and iCSs treated with 1-40 μM DOX (Fig. 4a). Peak plasma concentrations of DOX in patients are in the range 5-10 μM^42–45^. Statistical analyses showed a similar trend of response for hCSs and iCSs (Fig. 4a), with a statistically-significant increase at DOX concentrations within the therapeutic range. The concentration-dependent increase in DOX-mediated toxic effects was correlated with the number of CMs present in hCMs and iCMS, with almost a two-fold increase at 40 μM in hCSs (containing 1000 hCMs) versus an 8-fold increase in iCSs (containing 5000 iCMs) at the same concentration. The number of iCMs contained within iCSs is six-fold higher compared with the number of hCSs contained within hCSs. Data normalization against the number of CMs in the different spheroid types in Fig. 4a demonstrates that the increase in DOX–induced toxicity was similar between hCSs and iCSs. Hence, although the iCS have a greater representation of cardiomyocytes than the hCS, both appear to be similarly vulnerable to DOX-mediated toxicity.

Several studies have demonstrated a complex mechanism of DOX action on human heart tissue involving free radical formation, calcium dysregulation, mitochondrial dysfunction and topoisomerase II inhibition^46^. Reactive oxygen species (ROS) have been suggested to be responsible for most of the toxic effects in ECs in the human heart via eNOS uncoupling and peroxynitrous acid generation, with the latter arising from reaction of nitric oxide with superoxide^47^. To evaluate DOX-mediated intracellular NO synthesis, we measured DAF-FM diacetate fluorescence in both DOX-treated hCSs and iCSs (Fig. 4b). Our analyses demonstrated a statistically-significant increase in DOX-mediated NO synthesis starting at 10 μM in hCSs and 5 μM in iCSs. These differences probably arise from a lower level of NO formation by iCSs and their greater sensitivity to DOX due to the different cell ratios. L-NIO, a non-specific eNOS competitive antagonist, significantly alleviated DOX-mediated toxic effects in these cultures at concentrations above 10 μM (Fig. 4a). Interestingly, NOS inhibition by L-NIO did not rescue DOX toxicity in pure cardiomyocyte spheroids, whereas 2D cardiomyocyte monolayers, which were affected to a greater extent by DOX, showed a limited positive effect of L-NIO (Fig. S4).

**Figure 5:**
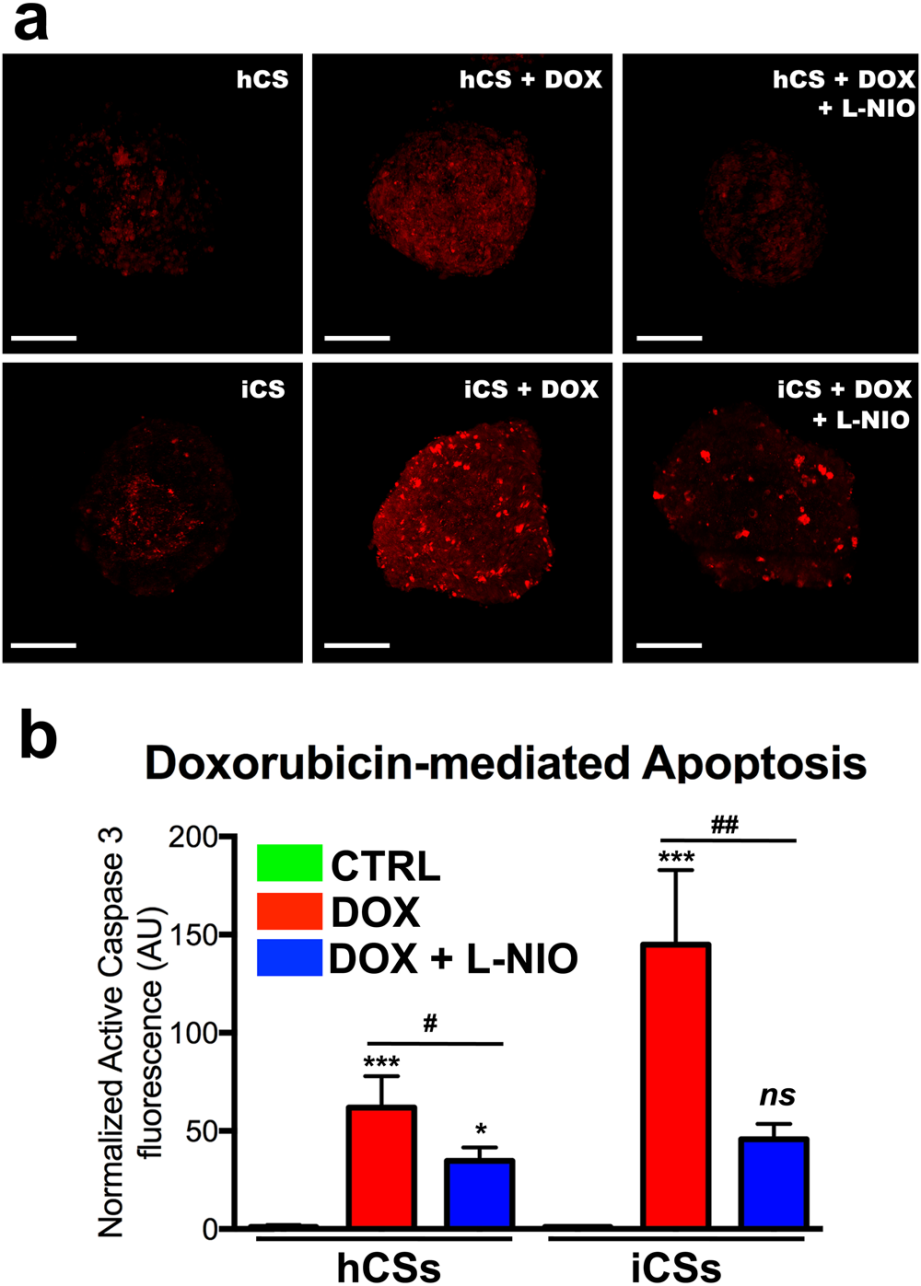
Doxorubicin-mediated apoptosis is inhibited by L-NIO. (**a**) Confocal analysis of hCSs and iCSs cultured with and without 20 μM DOX and 100 μM L-NIO and stained with antibodies against active caspase 3 (shown in red). (**b**) Statistical analysis (*n* = 3) of black-versus-white ratios of confocal images of hCSs and iCSs cultured and stained as described in panel (a). Data are presented as mean ± SEM. Scale bars: 200 μm. One-way ANOVA followed by Bonferroni’s multiple comparisons test. F (df 5, 12) = 1.153.

Confocal analysis of DOX-treated hCSs and iCSs stained with antibodies against active caspase 3 demonstrated that DOX-mediated apoptosis is inhibited in L-NIO-pre-treated spheroids (Fig. 5). In accordance with the data in Fig. 4, the extent of DOX-induced apoptotic effects in hCSs and iCSs appears to reflect the cell ratios used to generate the spheroids, and particularly their CM content.

Taken together, these data suggest that DOX-mediated toxicity and apoptosis in hCSs and iCSs is dependent on NO signalling.

### eNOS-mediated NO synthesis as a molecular target for DOX-induced toxic effects in the human heart

**Figure 6:**
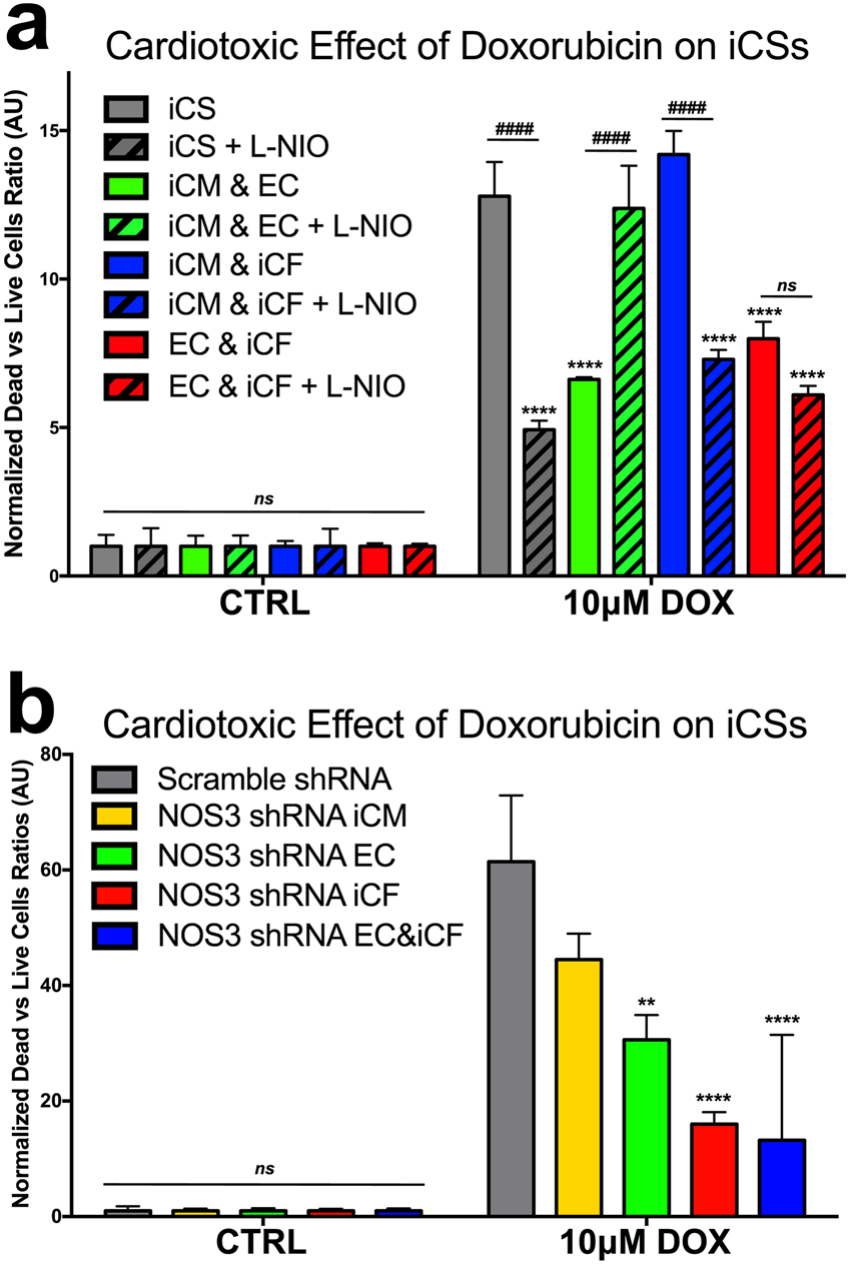
DOX toxicity is mediated via NOS in different cell types. (**a**) Comparison of DOX-mediated toxic effects in co-cultures from different cell populations (*n* = 6): (i) iCS generated from iCM, EC and iCF; (ii) iCMs combined with ECs only; (iii) iCMs combined with iCFs only; and iv) ECs combined with iCFs. 100 μM L-NIO was used to inhibit NOS activity. Two-way ANOVA followed by Tukey’s multiple comparisons test. F (df 7, 32) = 17.13. (**b**) Comparison of DOX-mediated toxicity in iCSs where eNOS was inhibited by using eNOS shRNA in specific cell types (*n* = 6). Scramble shRNA was used as control. ^*^Relative to iCS; ^♯^within the same cell population. Data are presented as mean ± SEM. Two-way ANOVA followed by Tukey’s multiple comparisons test. F (df 4, 40) = 5.846.

Based on the data demonstrating an NO-dependent DOX-induced toxic effect in iCSs, we examined the cell types responsible for this effect. To this end, we compared DOX-mediated effects using spheroids generated with only two out of the three cell types (iCMs, iCFs and ECs) (Fig. 6a). Statistical analysis demonstrated a protective role for ECs in co-cultures with either iCMs or iCFs after DOX treatment, whereas iCM-iCF co-cultures without ECs showed a similar response to DOX as observed in iCSs. The toxic action of DOX was restored by inhibition of NO synthesis with L-NIO in the iCM-EC, but not in the EC-iCF co-cultures further supporting the protective role of ECs and toxic effects of iCFs on iCMs, with CMs being the main target of damage.

In order to examine the molecular mechanisms of DOX-mediated toxic effects in iCSs, and the role of eNOS, one of the enzymes that constitutively generates NO in the heart, we stably silenced eNOS in either iCMs, or ECs or iCFs using NOS3 shRNAs prior to the formation of iCSs. Statistical analyses showed that silencing of eNOS in any cell type prevented DOX-mediated effects on iCSs at 10 μM, with the strongest inhibitory effect observed in NOS3-silenced iCFs, followed by ECs and then iCMs (Fig. 6b).

**Figure 7:**
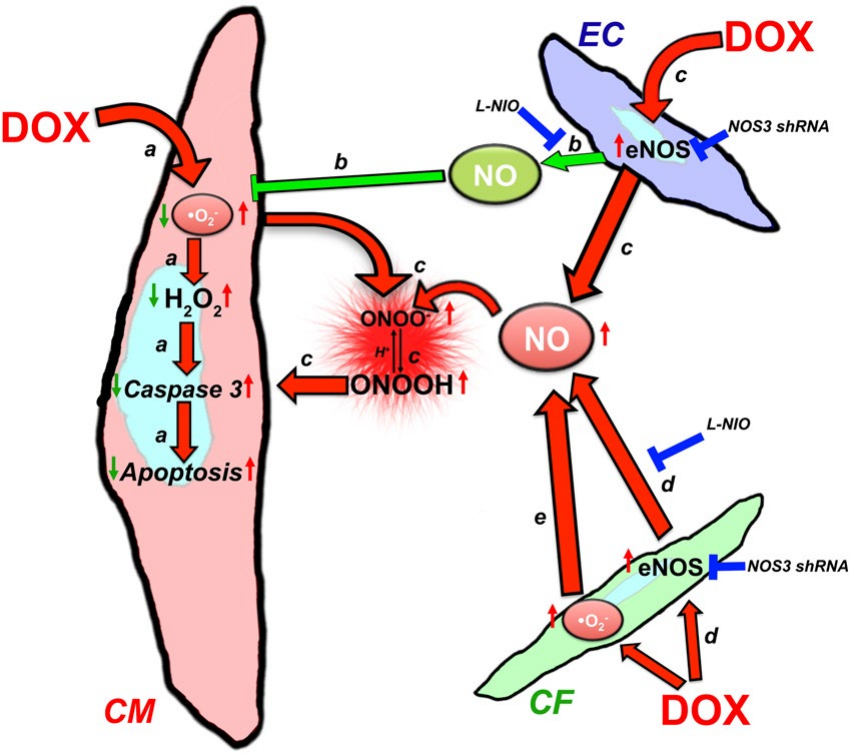
Cross-talk between different cell types in DOX-induced cardiotoxicity. (**a**) DOX-induced effects on cardiomyocytes occurs via ROS/caspase 3-mediated apoptosis. (**b**) EC-released NO protects cardiomyocytes against DOX-induced toxicity. (**c**) DOX-induced eNOS up-regulation in endothelial cells produces toxic NO species. (**d**) DOX-induced eNOS up-regulation in cardiac fibroblasts produces the majority of the toxic NO species. (**e**) DOX-induced cardiotoxic effects via ROS production in cardiac fibroblasts. At the molecular level, DOX/superoxide-induced apoptosis in cardiomyocytes can be either rescued by endothelial cell-released NO, via inhibition of a ROS-mediated toxic effect, or can be exacerbated by increased eNOS expression and peroxynitrous acid formation. Cardiac fibroblasts can trigger DOX/NO-toxic effects by overexpressing eNOS and increasing peroxynitrous acid generation, or could increase superoxide levels and therefore feedback to endothelial cell-mediated peroxynitrous acid and cardiomyocyte apoptosis.

Overall these data (summarized in Fig. 7) suggest that iCSs are suitable models to study toxic effects in a complex multicellular microenvironment such as the human heart. Additionally, it opens a new field of study based on the novel role played by iCFs in DOX-induced toxicity.

## Discussion

In this study, we have established for the first time the microenvironment or *niche* required to mimic the *in vivo* milieu of the human heart *in vitro*. This is based on direct analysis of cellular and extracellular features of the human heart *ex vivo*. Our data confirm that the ratio of different cell populations (CMs, CFs and ECs) is critical to successful modeling of the behaviour of the human heart. Adult CMs represent ~30% of the total cell population in a human heart, with a ratio of ECs and CMs equal to 3:1, even though the mass ratio of these two cell types is 0.04–0.05. In contrast, CFs represent ~ 60% of the cell population^12, 48–50^. To investigate the human heart properties *in vitro* we utilized human heart specimens to generate 3D *in vitro* models.

Generating hCSs from primary CMs of healthy and diseased specimens has great potential for disease modelling. However, based on our findings, primary CMs don’t survive in culture for extended periods without supporting cells. We therefore increased the ratio of CFs and ECs for the generation of hCSs with a final ratio of six CFs to three ECs and one CM. However, human heart specimens are not commonly available, which limits their use in generating *in vitro* models for high throughput assays. For this reason, we investigated whether CSs could be generated from commercially available iCMs. There are clear limitations associated with the use of iPSC-CMs containing iCSs, as these cells retain an embryonic/neonatal phenotype compared to primary cells (e.g. spontaneous beating in culture^11^). We therefore used different cell ratios for iCSs, to better mimic observed *ex vivo* cell populations by co-culturing two iCMs, one EC and one CF, reflecting the differences in cell size and ratios present in young versus adult human hearts.

The vascular network observed in hCSs and iCSs reflects the ratio of cells used. Compared to hCSs, the vascular networks in iCSs followed the path generated by ECs and CFs (Fig. 2), where these cells aggregated in multiple areas and from which several ramifications form (Fig. S3). Interestingly, CFs appear to play a supportive role for the vascular network formation within iCSs, by providing the required ECM scaffold for *ex novo* microvascular network formation from single ECs (Fig. S3). The only other attempt to evaluate the possible advantages of co-culturing iCMs with ECs and CFs is from Ravenscroft *et al*.^51^ who demonstrated that co-cultured microtissues better replicate the biochemical and physiological properties of the human heart compared to iCMs alone. Beside the differences in the kinetics of formation of the cardiac microtissues generated in this previous study and our iCSs (two weeks versus four days, respectively), these two models differ in other aspects. Thus, they are generated using different culturing conditions (non-adherent plates versus hanging drop cultures), but more importantly the cardiac microtissues lack the microvascular network formation observed in our iCSs (Fig. S3). This could be explained by the differences in the ratios and total numbers of cells used. Morphological differences between these two 3D *in vitro* models may also affect their behavior. For instance, apoptosis typically occurs in the center of any tissue with a radius bigger than 200 μm in the absence of a preformed vasculature^12, 52^. Our gene profiling data demonstrate that iCSs are similar to human LV specimens (data not shown, provided for review in supplementary information). This would affect the dose response to pharmacological agents acting on the heart. In order to evaluate the use of iCSs as a model to predict toxicological effects on the human heart, we treated them with DOX, a well-known cardiotoxic agent. DOX-induced oxidative stress and cardiotoxicity has been suggested to occur via increased endothelial nitric oxide synthase (eNOS) expression and activity^22–25^.

Previously reported mechanisms for DOX-mediated cardiotoxic effects detected with a cumulative dose of 100-600 mg/m^2^, include lipid peroxidation, mitochondrial dysfunction and apoptotic pathways, with these effects dramatically limiting its use as a chemotherapeutic agents^26–29^. A severe late-onset dilated cardiomyopathy (more than one year after completion of treatment) is often observed in DOX-treated young patients with a dose > 250 mg/m^2^ ^24, 53, 54^, and in both overweight^55^ and other adults at a cumulative dose > 450 mg/m^2^ ^56^. Among the proposed mechanisms, reactive oxygen species (ROS) seem to play a mayor role^30–35^.

At low physiological concentrations NO plays a protective role in the heart where it is constitutively synthesized by eNOS in both CMs and ECs^21^. It is important to note that human CMs express all NOS isoforms (eNOS, iNOS and nNOS), whereas ECs express both eNOS and iNOS, but not nNOS. Few previous reports have demonstrated eNOS expression in CFs, possibly due to the culture conditions and cell stage^57–61^. Interestingly, Ravenscroft *et al*.^51^ also measured increased levels of eNOS in CFs compared to CMs.

Kaliventhi *et al*.^62^ showed that both chemical and genetic inhibition of eNOS in ECs exacerbated DOX-induced ROS production, providing evidence for cross-talk between ROS and NO synthesis in ECs. It is therefore possible that it is the combination of DOX-induced ROS and NO syntheses that mediates toxic effects via peroxynitrous acid generation.

The data obtained here for the 3D spheroids of either iCMs alone, or these cells together with other supporting cells, indicate that NO from ECs plays a protective role in CMs from DOX, probably via eNOS uncoupling. Similar to our data (Fig. 6), Kalivendhi *et al*.^62^ have shown that DOX treatment increases eNOS transcription and protein activity in BAECs, and DOX-induced apoptosis can be rescued by antisense eNOS mRNA. Similarly, Kalivendhi *et al*.^62^, showed that pharmacological inhibition with a competitive antagonist of eNOS increased DOX-induced apoptosis, as seen in co-cultured HCAECs/iPSC-CM spheroids. Our data for iCSs confirm the protective role played by NO from ECs following DOX treatment, together with its mediation of toxic effects by increasing peroxynitrous acid generation and caspase 3 activation (Fig. 7).

Our study has identified, for the first time, the toxic role played by cardiac fibroblasts in DOX-induced apoptosis of cardiomyocytes via NO. Similar to the effect observed using iCFs in iCSs, DOX has previously been shown to induce caspase 3-mediated apoptosis in mouse embryonic fibroblast cultures via ROS production in a more potent manner compared to H_2_O_2_^36^. Prior to our study, it was not clear whether DOX-induced toxic effects in these cells occured upstream of eNOS, or via other isoforms such as iNOS^38^. Our data support a role for eNOS in CFs to mediating DOX-induced toxic effects on CMs, based on our chemical and genetic inhibition of eNOS in these cells (Fig. 7). Interestingly, NOS inhibition by L-NIO did not protect against the DOX toxicity in the iCMs in 3D cultures, whereas 2D iCMs monolayer cultures, which were more markedly affected by the drug, showed a limited positive effect of L-NIO (Fig. S4).

These findings suggest an alternative mechanism to target DOX/ROS-mediated cardiotoxic effects involving eNOS^37^. As DOX is a first choice agent to treat several cancers, this novel mechanism of DOX-mediated cardiotoxicity via CFs, opens a new field of investigation for inhibiting cardiomyopathies in cancer patients. Our study also highlights the advantages of the co-culture approach compared to current cell culture practices, as CSs represent a more reliable and nuanced model of the behavior of the human heart with regard to cardiotoxic effects, and also for the study of basic and translational biology and physiology.

Possible further applications for iCSs include their use as building blocks for 3D bioprinting thanks to their pre-formed vascular network and capacity to undergo self-assembly. These properties make them suitable for generation of bioengineered models of the human heart of both increased size and complexity, including studies on blood flow and mechanical stimuli^12, 52^.

## Methods

### Drugs and reagents

Primary antibodies: Mouse monoclonal antihuman CD31/PECAM was purchased from BD Pharmingen (San Diego, CA, USA). Mouse monoclonal anti-α-actinin (Sarcomeric) was purchased from Sigma-Aldrich (St. Louis, MO, USA). Mouse monoclonal [1C11] anti-human Cardiac Troponin T (Texas Red^®^), mouse monoclonal [A17] to fibronectin, rabbit polyclonal to laminin, rabbit polyclonal to collagen type IV, mouse monoclonal [V9] to vimentin (Alexa Fluor^®^ 488) and rabbit polyclonal to active caspase-3 antibodies were purchased from Abcam (Cambridge, MA, USA). Secondary donkey anti-rabbit and anti-mouse secondary fluorochrome-conjugated antibodies were from Jackson Immunological Research Labs, Inc., West Grove, PA, USA. NucBlue® Live ReadyProbes® Reagent (Hoechst 33342) was purchased from Invitrogen (Carlsbad, CA, USA). Extracellular matrix: Fibronectin from bovine plasma was purchased from Sigma-Aldrich (St. Louis, MO, USA). Drugs: L-NIO (N5-(1-iminoethyl)-L-ornithine, dihydrochloride, used at 100 μM) was purchased from Cayman Chemical (Ann Arbor, MI, USA). Doxorubicin hydrochloride (used at 1, 5, 10, 20 and 40 μM) was purchased from Sigma-Aldrich (St. Louis, MO, USA).

### Cells and cardiac spheroid formation

Human primary cardiomyocytes (hCMs) were isolated from human heart sections of left ventricular myocardium from healthy unused donor hearts that were first thawed from liquid nitrogen to 37 °C and then digested enzymatically. Human specimens were kindly obtained from the Sydney Heart Bank at the University of Sydney. Human tissue was used in accordance with the ethical guidelines of the University of Sydney Human Research Ethics Committee, from which Human Research Ethics Committee approval was previously obtained (Project Title: The Sydney Human Heart Tissue Bank (SHB), Project No: 2012/2814). Signed patient consent was obtained for all samples in this tissue bank. For cardiomyocyte isolation, 300 μm frozen sections were digested at 37 °C in Krebs buffer (120 mM NaCl, 4.7 mM KCl, 25 mM NaHCO_3_, 1.2 mM KH_2_PO_4_, 1 mM MgSO_4_, 1.2 mM CaCl_2_, 12 mM glucose, 100 μM ascorbic acid, pH = 7.4) containing 1 mg/ml collagenase B (Roche, Basel, CH), 1 mg/ml collagenase D (Roche, Basel, CH) and 30 mM 2,3-butanedione monoxime (BDM) for 20 min under agitation. Sections were then mechanically dissociated using a pipet and filtered using a 200 μm mesh to collect single cells and remove undigested tissue. Viability of rod-shaped striated cardiomyocytes was evaluated using Trypan Blue staining.

Cor.4U induced pluripotent stem cell-derived cardiomyocytes (iCMs) and fibroblasts (iCFs) were obtained from Axiogenesis (Cologne, Germany). Human coronary artery endothelial cells (HCAECs) were purchased from Cell Applications, Inc (San Diego, CA, USA). Cells were plated and cultured according to supplier’s instructions. iCMs and iCFs were cultured in Cor.4U Culture Medium (Axiogenesis), whereas HCAECs were cultured in Human MesoEndo Cell Growth Medium (Cell Applications, Inc).

Human cardiac spheroids (hCSs) were generated by co-culturing 1000 hCMs (immediately after their isolation) with 3000 ECs and 6000 iCFs in 40 μl hanging drop cultures, whereas induced pluripotent cardiac spheroids (iCSs) were generated by combining 6000 iCMs together with 3000 ECs and 3000 iCFs in 40 μl hanging drop cultures using the Perfecta 3D® 96 well Hanging Drop Plates (3D Biomatrix, Ann Arbor, MI, USA). Single or two cell type spheroids were generated in hanging drop cultures using: i) 6000 iCMs alone; ii) 6000 iCMs plus 3000 ECs; iii) 6000 iCMs plus 3000 iCFs; iv) 3000 ECs plus 3000 iCFs. Drug treatment was conducted following 1 day (hCSs) or 4 days (iCSs) in hanging drop cultures in the same well for 24 h. To allow tissue penetration, L-NIO was added 1 h prior to DOX.

### Tissue and spheroid immunolabeling and confocal imaging

Spheroids were fixed in 4% paraformaldehyde for 60 min and permeabilized in phosphate buffered saline/0.01% sodium azide (PBSA) containing 0.02% Triton-X-100 (60 min). Tissue slices and spheroids were then blocked with a 3% bovine serum albumin (BSA)/PBSA solution, and then incubated with appropriate primary (15 μg/ml) and secondary (10 μg/ml) antibodies overnight at 4 °C. Tissues and spheroids were incubated with antibodies against cTNT, CD31 and vimentin, to stain hCMs/iCMs, ECs and iCFs, respectively. DNA Hoechst stain for nuclei labelling (Invitrogen, Carlsbad, CA, USA) was added together with the secondary antibody. Specimens were mounted using Vectashield^®^ Mounting Medium (Vector Laboratories, Burlingame, CA, USA). Fluorescent imaging used a Zeiss LSM 510 Meta Spectral Confocal Microscope (Carl Zeiss AG, Oberkochen, Germany). Optical sectioning along the Z axis was performed and the images collapsed into a single focal plane using the manufacturer’s software. Images were processed using NIH Image 1.47 v software (National Institutes of Health, Bethesda, MD, USA) and Adobe Photoshop CS5 (Adobe Systems, Inc., San Jose, CA, USA). For 3D reconstructions, images were processed by Imaris V 7.6 (Bitplane, Concord, MA, USA).

### Toxicity assay

Monolayer cultures of iCMs were plated and CSs were transferred in 96-well clear bottom black polystyrene microplates (Corning®, New York, NY, USA) 24 hours prior to their treatment with DOX and/or L-NIO. Live/Dead® Viability/Cytotoxicity Kit for mammalian cells (Invitrogen, Carlsbad, CA, USA) was used according to manufacturer’s instructions to evaluate toxicity, calculated as a ratio between dead versus live cells, measured in each well at 645 nm (for ethidium homodimer) and 530 nm (calcein-AM), respectively. Ratios were normalized against the number of cells present within the spheroid and against the control culture (untreated spheroids in case of hCSs and iCSs, iCSs at the different DOX concentrations in case of comparing studies). GraphPad Prism™ (La Jolla, CA) was used for data analysis and statistics.

### Apoptotic assay

Fixed spheroids were exposed sequentially to the primary active caspase 3 antibody (15 μg/ml) and secondary (10 μg/ml) antibodies (both overnight, 4 °C), and then stained with Hoechst stain. They were imaged using a Zeiss LSM 510 Meta Spectral Confocal Microscope (Carl Zeiss AG, Oberkochen, Germany) and active caspase 3-positive area over total area were measured using a black-to-white ratio by using NIH ImageJ 1.47v software (National Institutes of Health, Bethesda, MD, USA) and Adobe Photoshop CS6 (Adobe Systems, Inc., San Jose, CA, USA). Active caspase 3 measurements were normalized against the number of cells and against the control (untreated spheroids) and then analyzed using GraphPad Prism™ (La Jolla, CA).

### Intracellular NO synthesis

Intracellular NO was detected by using DAF-FM diacetate (Invitrogen, Carlsbad, CA, USA) as previously described^63^. Briefly, hCSs and iCSs were treated in presence of DOX with or without L-NIO for 24 h in hanging drop plates. Spheroids were then transferred in clear bottom black 96 well plates by centrifugation. Spheroids were rinsed twice with PBS and 1 μM DAF-FM diacetate solution was prepared freshly in PBS. After 30 min, DAF-FM diacetate solution was removed, spheroids were rinsed twice with PBS and fresh PBS was added for 30 min. For measurements of NO synthesis, the microplate was placed in the FlexStation® 3 Multimode Plate Reader (Molecular Devices, Sunnyvale, CA, USA) and measurements were recorded at 515 nm. NO synthesis was calculated by normalizing measurements against total number of cells and then against control (media only without DOX and/or L-NIO), and analysed using GraphPad Prism™ (La Jolla, CA).

### eNOS Silencing

iCMs, ECs and iCFs were cultured separately in T75 flasks and transfected with either NOS3 or scrambled shRNA lentiviral particles (SantaCruz Biotechnology, Dallas, TX, USA). Both shRNA lentiviral particles were diluted in media containing Polybrene (SantaCruz Biotechnology, Dallas, TX, USA) to give a final concentration of 1% (v/v) of lentiviral particles. Cells were incubated with transfection solution at 37 °C, under 5% CO_2_ for 24 h prior to their use for iCS generation.

### Statistical analysis

Experimental data are expressed as the mean ± SEM from at least three independent experiments. ANOVA followed by Bonferroni post hoc test was performed using GraphPad Prism™ (La Jolla, CA).

## Electronic supplementary material

Supplementary Information

Supplemental Video 1

Supplemental Video 2
