# Supplementary material for: Cardiac spheroids as promising in vitro models to study the human heart microenvironment
Source: Sci Rep. 2017 Aug 1;7:7005. doi: 10.1038/s41598-017-06385-8 (PMC5539326; doi:10.1038/s41598-017-06385-8)
Supplement: Supplementary file 1 — Supplementary Information [file 41598_2017_6385_MOESM1_ESM.pdf]

**Title: Cardiac spheroids as promising *in vitro* models to study the human heart microenvironment.**

**Authors:** Liudmila Polonchuk[1], Mamta Chabria[1], Laura Badi[1], Jean-Christophe Hoflack[1], Gemma Figtree[2], Michael J. Davies[3], Carmine Gentile[2,4,5*].

[1]Roche Pharma Research and Early Development, Roche Innovation Center Basel, F. Hoffmann-La Roche Ltd., 4070 Basel, Switzerland.

[2]Sydney Medical School, University of Sydney, Sydney, 2000, Australia

[3]Department of Biomedical Sciences, Panum Institute, University of Copenhagen, Copenhagen 2200, Denmark.

[4]Heart Research Institute, Newtown, 2041, Australia.

[5]Beth Israel Deaconess Medical Center, Harvard Medical School, Boston, USA.

*Corresponding author: carmine.gentile@sydney.edu.au

**Methods**

**Electrical activity measurements related to Cells and Cardiac Spheroid Formation methods**

A 300μm slice of human heart tissue was placed on top of the 64-multielectrode array of the MEA2100-System (MEA, MultiChannel Systems, Germany). External field stimulation was applied to trigger spontaneous activity recorded from the tissue before or after adding 10mM isoproterenol.

**TEM related to Cells and Cardiac Spheroid Formation methods**

hCSs were fixed with 2.5% glutaraldehyde in 0.1M phosphate buffer for one hour first, rinsed three times with 0.1M phosphate buffer (5 min each) and then fixed with 1% osmium tetroxide in 0.1M phosphate buffer for 60 min. Fixed spheroids were rinsed three times with Milli-Q water and then dehydrated with increasing concentrations of ethanol (30, 50, 70, 95 and 100%). Resin was added with increasing concentrations between 25, 50, 75 and 100% by replacing the solution every 3 h in fume hood. Specimens were embedded in polyethylene BEEM® capsules in 60°C oven overnight. Semi-thin sections (500nm thick) were cut, stained with toluidine blue, and observed under a conventional microscope. Ultrathin thin sections (90 nm) were cut using a microtome and collected on copper mesh grids. Sections were first stained with 2% uranyl acetate, rinsed with Milli-Q water, stained with lead citrate and finally rinsed with Milli-Q water. Images were acquired using a 120 kV TEM JEOL 1400 (JEOL, Tokyo, Japan).

## Supplementary Figures

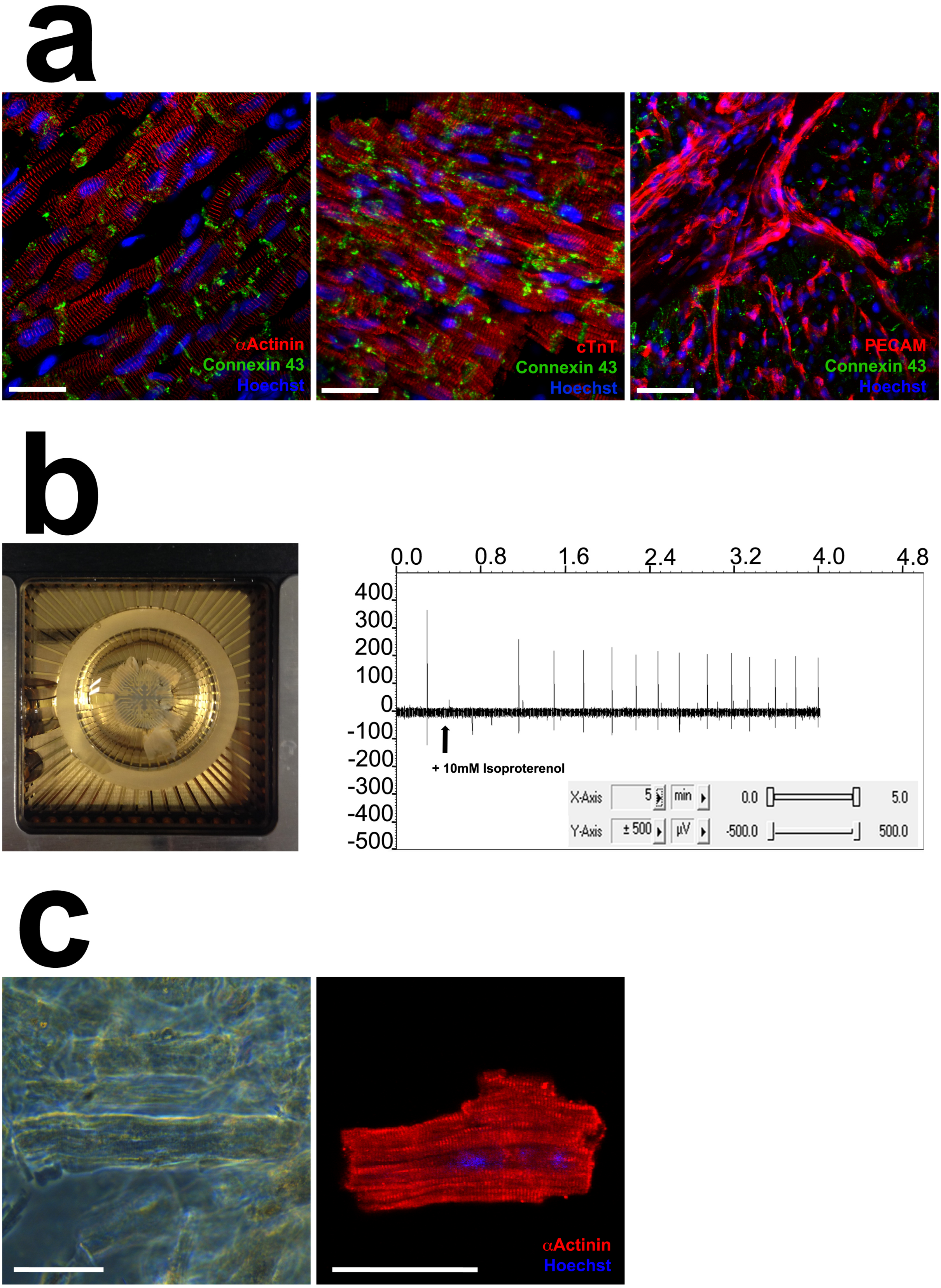


**Figure S1** related to Figure 1.

Isolation of cardiomyocytes from human heart sections. **(a)** Confocal analysis of *ex vivo* human heart tissue stained with antibodies specific for CMs (cardiac Troponin T or cTNT, sarcomeric α-actinin and connexin 43) and ECs (PECAM). Nuclei are stained with Hoechst stain. **(b)** Field potential measurements in a human heart section using a microelectrode array (MEA) system. As shown in (b), regular 1 Hz spontaneous electrical activity is recorded ~4 min after addition of 10mM isoproterenol. **(c)** Brightfield image of isolated human cardiomyocytes and confocal analysis of an isolated human cardiomyocytes stained using antibodies against sarcomeric α-actinin. Nuclei are stained with Hoechst stain (blue). Scale bar: 50μm.

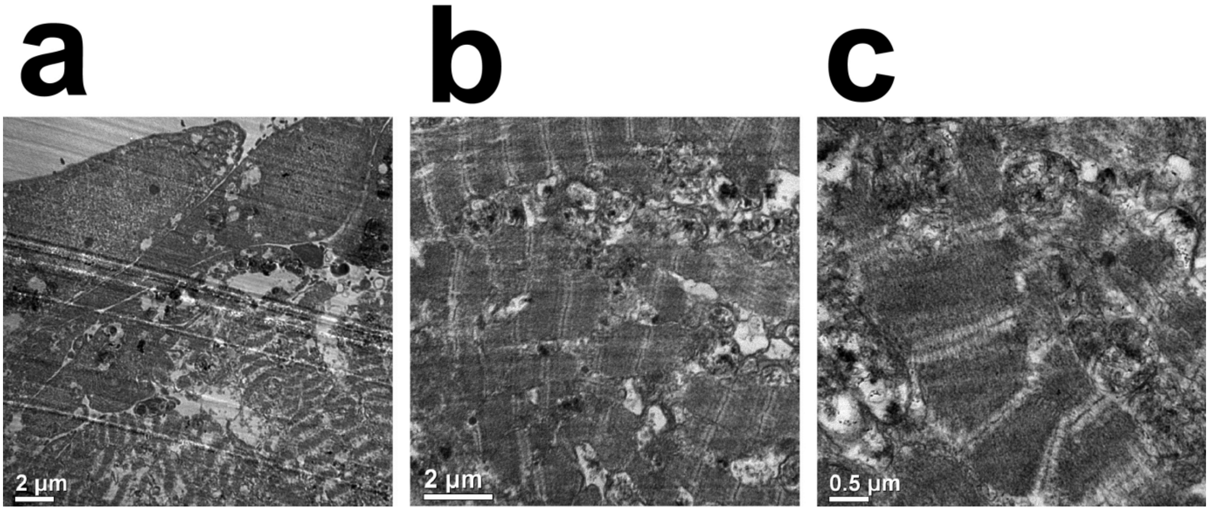


**Figure S2** related to Figure 1.

**(a-c)** Transmitted electron microscopy (TEM) micrographs of hCSs showing intact sarcomeres and mitochondria following three days in hanging drop cultures. hCMs sort out within hCS, whereas iCFs are present in the outer layer **(a)**. Lower **(b)** and higher **(c)** magnification images show structural appearance of sarcomeres and mitochondria within hCSs.

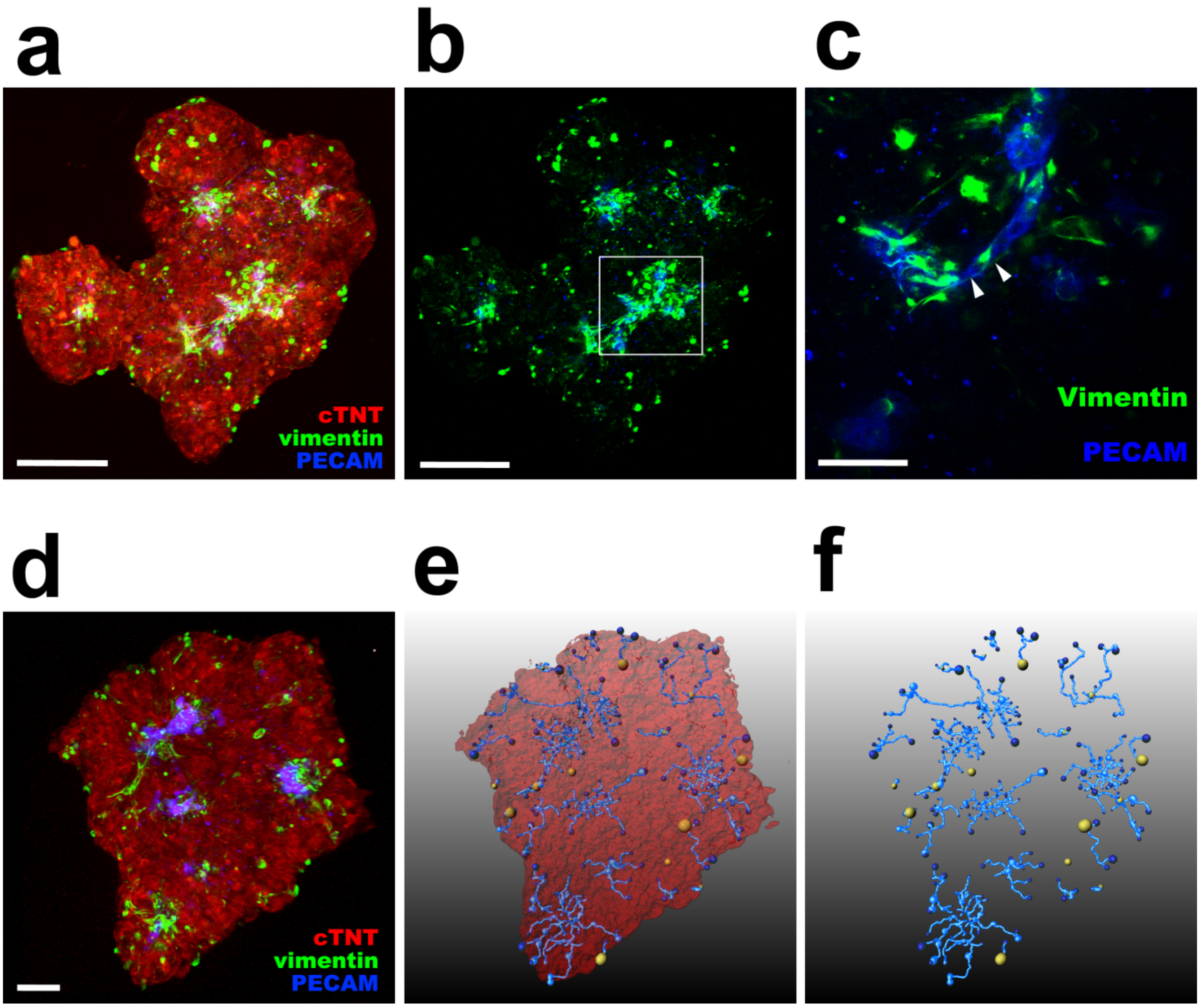


**Figure S3** related to Figure 2.

The role played by cardiac fibroblasts in iCS formation. **(a-c)** Low **(a and b)** and high **(c)** magnification images of a forming iCS stained with antibodies against vimentin (green), PECAM (blue) and cTNT (red, in **(a)** only). **(c)** is a subset of Z-stack composing the insert in panel **(b)**, showing how vimentin-positive iCFs facilitate EC network formation and spheroid formation by pulling together smaller spheroids and wrapping around ECs, as highlighted by the arrowheads in **(c)**. **(d-f)** Three-dimensional analysis of vimentin-positive iCFs shows a network that irradiates from several centers within a iCS. **(d)** Confocal image of an iCS stained with antibodies against vimentin (green), PECAM (blue) and cTNT (red). 3D Imaris reconstruction of the iCS depicted in **(d)**, showing vimentin-positive iCF network with **(e)** and without (**f**, blue “sticks”) iCMs. Yellow spheres depict multiple starting-points of the fibroblast

network within a iCS, whereas blue spheres indicate end-points of the same network. Scale bars: 200μm in **(a)** and **(b),** 50μm in **(c)** and 100μm in **(d)**.

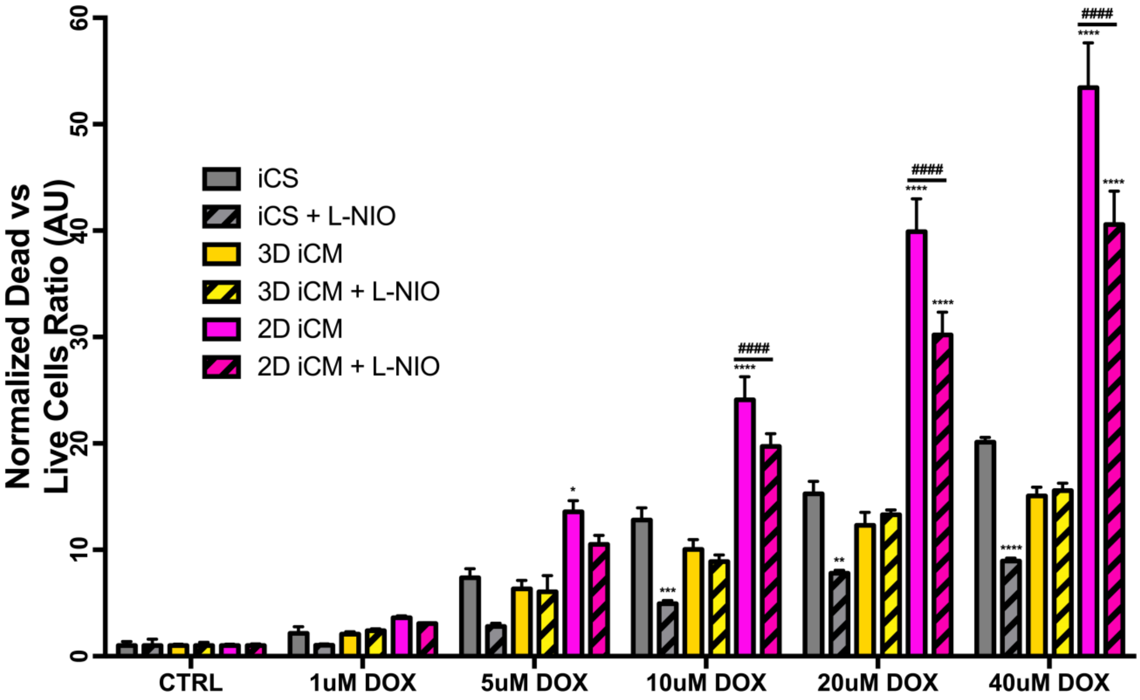


**Figure S4** related to Figure 4.

DOX-mediated cardiotoxicity via NOS activity in 2D versus 3D cultures. Statistical analysis (n=3) of DOX-mediated toxic effects evaluated as a ratio between dead and live cells following 24h of treatment. 100μM L-NIO was used to inhibit NOS activity. * relative to iCS; # within the same group. Two-way ANOVA followed by Tukey's multiple comparisons test. F (df 25, 72) = 27.86. Data are presented as mean ± SEM.

**Video S1 and S2** related to Figure 1. Spontaneously beating iCSs in hanging drop culture. Video S1 shows a completely spheroidal beating iCSs, whereas Video S2 shows a beating heart-shaped iCS.
